# Spina bifida cystica and severe congenital bilateral talipes equinovarus in one twin of a monoamniotic pair: a case report

**DOI:** 10.1186/s13104-017-3108-5

**Published:** 2017-12-28

**Authors:** Benjamin Momo Kadia, Desmond Aroke, Frank-Leonel Tianyi, Ndemazie Nkafu Bechem, Christian Akem Dimala

**Affiliations:** 1 Foumbot District Hospital, Foumbot, Cameroon; 2 Grace Community Health and Development Association, Kumba, Cameroon; 3 Health and Human Development (2HD) Research Network, Douala, Cameroon; 4 Mbengwi District Hospital, Mbengwi, Cameroon; 5 Mayo Darle Sub-Divisional Hospital, Mayo Darle, Cameroon; 6 School of Health Sciences, University of Nottingham, Nottingham, UK; 7 Faculty of Epidemiology and Population Health, London School of Hygiene and Tropical Medicine, London, UK; 8 Department of Orthopaedics, Southend University Hospital, Essex, UK

**Keywords:** Monoamniotic, Spina bifida, Talipes equinovarus, Discordant, Case report

## Abstract

**Background:**

Spina bifida and congenital talipes equinovarus (CTEV) are common congenital malformations which may occur together and increase morbidity. Monozygous twins are particularly at risk of these malformations and discordance in one type of malformation is typical. The occurrence of both spina bifida and CTEV in one twin of a monozygotic pair is rare.

**Case presentation:**

A 22 year-old Cameroonian primigravida at 36 weeks of a twin gestation was received in our district hospital at the expulsive phase of labour on a background of sub-optimal antenatal care. A caesarean section indicated for cephalo-pelvic disproportion was performed and life monoamniotic male twins were extracted. The first twin was normal. The second twin had spina bifida cystica and severe bilateral CTEV. Routine postnatal care was ensured and at day 2 of life, the affected twin was evacuated to a tertiary hospital for proper management. He was later on reported dead from complications of hydrocephalus.

**Conclusions:**

Spina bifida cystica with severe bilateral CTEV in one twin of a monoamniotic pair illustrates the complexity in the interplay of causal factors of these malformations even among monozygotic twins who are assumed to share similar genetic and environmental features. The occurrence and poor outcome of the malformations was probably potentiated by poor antenatal care. With postnatal diagnoses, a better outcome was difficult to secure even with prompt referral. Early prenatal diagnoses and appropriate counseling of parents are cardinal.

## Background

Congenital talipes equinovarus (CTEV) is the most common congenital disorder affecting the musculoskeletal system [1]. Its incidence varies from 1 to 6 per 1000 live births depending on racial differences [2]. It has a male preponderance and is bilateral in about 50% of cases [2, 3]. In most newborns the limb deformity occurs in isolation (idiopathic CTEV). In a minority of neonates, CTEV occurs in association with other congenital abnormalities of which a considerable proportion are neural tube defects, notably, spina bifida [4]. Spina bifida is caused by failure of fusion of the vertebral arches and possibly the underlying neural tube. In spina bifida occulta, the meninges herniate through the bony defect and are covered by skin while in spina bifida cystica the roof of the defect is formed by exposed neural tissue. Spina bifida is found in about 4.4% of newborns with CTEV [5] and in such cases there is an associated increased morbidity.

From a hereditary perspective, the process of twinning has been implicated in the aetiology of malformations such as spina bifida and CTEV [6]. Monozygotic twins are particularly at risk of these malformations which may occur discordantly. However, previous reports typically describe discordance in one type of malformation [7, 8]. The occurrence of both spina bifida and CTEV in one twin of a monozygotic pair is rare. In this report, we present such an unusual case and we discuss possible aetiologic factors and prognostic challenges.

## Case presentation

A 22 year-old Cameroonian primigravida at 36 weeks of gestation was received in our district hospital for labour type pain for about 17 h. This was associated with continuous and abundant flow of clear fluid per vagina. She had started antenatal care visits at 19 weeks of gestation at a health center and she started receiving folic acid supplementation from then. Her immunization status was up-to-date. Her past history was negative for alcohol or tobacco consumption and she had no known family history of foetal anomalies. According to her past records, only one obstetric ultrasound scan was done at 19 weeks of gestation at a secondary hospital and had revealed a monoamniotic twin gestation with no apparent structural or growth abnormalities. Other routine antenatal laboratory investigations were unrevealing.

**Fig. 1:**
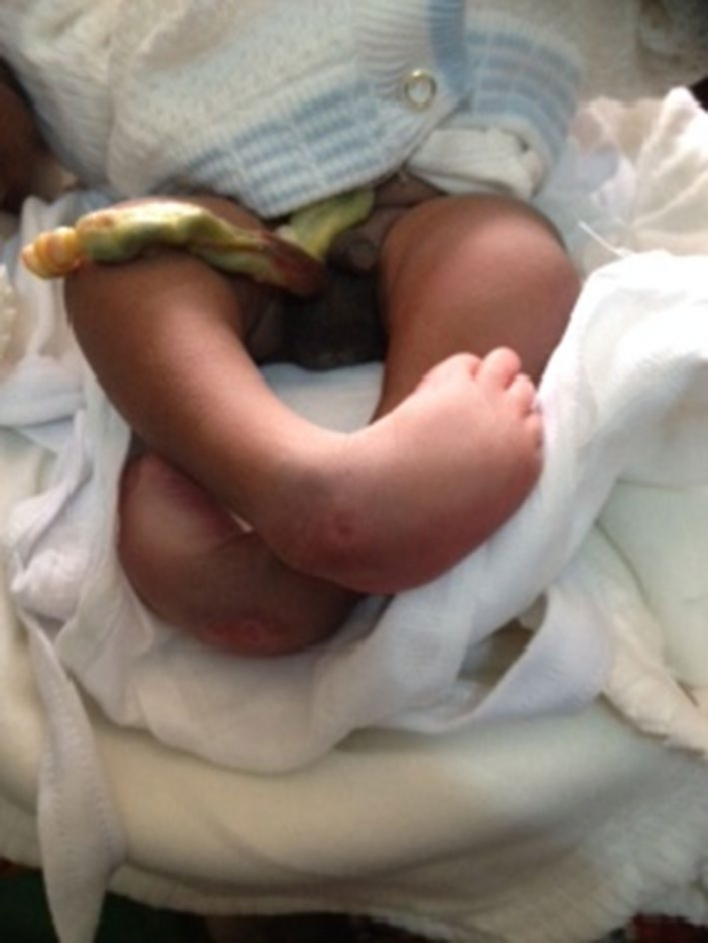
Bilateral talipes equinovarus in the second twin

**Fig. 2:**
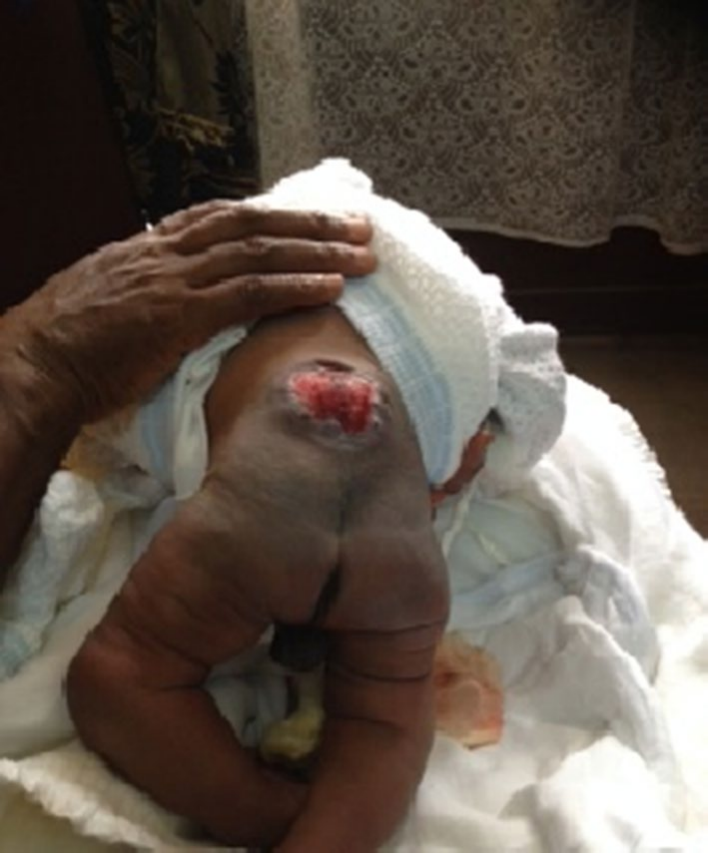
Spina bifida cystica in the second twin

Obstetric examination revealed that she had presented at the expulsive phase of labour with the first twin’s presentation being cephalic. Clinical pelvimetry was suggestive of cephalo-pelvic disproportion. A caesarean section was performed and life male monoamniotic-monochorionic twins were extracted. The first twin had APGAR scores of 8 and 9 at 1 and 5 min and weighed 2800 g. The second twin had APGAR scores of 7 and 8 at 1 and 5 min and weighed 2724 g. The physical examination of the first twin was normal. The second twin had symmetrically deformed feet: the hindfeet were in equinus and varus, there was cavus of the midfeet and the forefeet were adducted and inverted (Fig. 1). In addition, the muscle tone in the neonate’s lower extremities was low. A defect in the lumbar spine through which there was considerable protrusion and exposure of the spinal cord and its coverings was also observed (Fig. 2). The Pirani score was used to grade the severity of the limb deformity in the second twin: the midfoot score was 2.5/3 (curved lateral border: 1, medial crease: 1, and talar head coverage: 0.5), and the hindfoot score was 2/3 (posterior crease: 1, rigid equinus: 0.5, and empty heel: 0.5) giving a total of 4.5/6. These findings were indicative of severe bilateral TEV in the second twin. No other abnormalities were noted on examination of the second twin.

It was concluded that the twins were discordant for spina bifida and bilateral CTEV. Routine postnatal care was ensured and at day two of life the affected neonate was referred to a tertiary hospital for proper management. At the referral site, surgical repair of the spina bifida defect was planned. However, before the surgical procedure could be carried out, it was reported that the neonate had developed hydrocephalus and had died from its complications.

## Discussion and conclusions

Congenital abnormalities whether concordant or discordant occur in approximately 10% of monozygotic twins. Monozygotic twins, having their origin from the same fertilized ovum, are considered genetically identical [9, 10] and they have a higher risk of concordant congenital abnormalities when compared with other twins [3, 10]. Nonetheless, previous reports suggest that the least asymmetries in physiological, environmental or mechanical events during intrauterine development may result in phenotypic differences among these twins [11]. The severe grades of the malformations in the second twin in our report makes the case perplexing especially because of the lack of previous reports for comparisons. Importantly, low-income settings possess limited resources to explore the possibility of other associated anomalies in such discordant twin pairs.

Congenital talipes equinovarus and spina bifida can be diagnosed antenatally or postnatally. Antenatal diagnosis can be conveniently done with ultrasound scan although there is a wide variation in the reported accuracy [5]. In the current report, the obstetric history revealed that an ultrasound scan was done at 19 weeks of gestation. However, there may be a transient deformity in the foetal limb in the early weeks of gestation resembling CTEV and therefore later scans are more reliable for the diagnosis [12]. Furthermore, previous reports suggest that in the course of pregnancy, the complexity of CTEV could change from isolated CTEV to CTEV associated with other abnormalities like spina bifida [13]. In our case, the lack of serial and reliable obstetric ultrasound scans significantly limited the appraisal of the sequence of development of the malformations in the second twin even though it is likely that CTEV was due to spina bifida. Observer bias leading to misdiagnosis on antenatal ultrasound cannot be underestimated as a limitation to antenatal diagnosis in a resource-limited setting.

In the current report, postnatal diagnoses of congenital malformations were made via clinical examination. With regards to CTEV, diagnosis on clinical examination is straightforward and is made on the basis of irreducible equinus, varus of the hindfoot, adduction of the forefoot, cavus, and an “empty” heel pad [6, 14]. Clinical examination allows for proper assessment of the degree of deformity contrary to prenatal diagnosis [15]. The degree of deformity could be assessed using the methods described either by Pirani or Dimeglio but the Pirani score is widely utilized because of its simplicity [5]. As concerns spina bifida, the condition is usually obvious at birth especially when it is spina bifida cystica.

Irrespective of the severity of CTEV revealed by the Pirani score, the overall prognosis of the affected twin was expected to be contingent on the management of the open spina bifida, which was likely at the origin of the deformed limbs. Open spina bifida is the most common type of spina bifida associated with brain defects and about 90% of cases with these brain defects have Chiari malformation type II [16]. This malformation involves the herniation of the cerebellum upward into the middle fossa and downward into the cervical spinal canal [17]. Subsequent compression of the respiratory centre and upper airway dysfunction lead to respiratory failure [18]. Chiari malformation type II and aqueductal stenosis are major factors implicated in the development of hydrocephalus in neonates with open spina bifida [19]. The incidence of hydrocephalus in open spina bifida usually varies between 15 and 25% [20] but rates of up to 40% have been reported [21]. Other feared complications of open spina bifida include spinal cord injury and contamination of cerebrospinal fluid and meningitis [22].

With respect to the antenatal history in the case presentation, the late start of folic acid supplementation is worth discussing. Lack of early or sufficient folic acid supplementation in pregnancy has been implicated in the development of congenital malformations including spina bifida and CTEV [23]. Even though there is much controversy on the role of folic acid supplementation in the prevention of these malformations [23], it has been reported that monochorionic twins are more likely to intensely compete for resources in utero and this may favour discordance in malformations particularly if the supplementation of a nutrient like folic acid is inadequate. In line with a previous report by Machin et al., the intrauterine environment may have been unequal for the twins in our case, possibly because of the number of cells allocated to each twin, the timing of the twinning process, and the vascular distribution from the placenta [24].

In conclusion, spina bifida cystica and severe bilateral CTEV in one twin of a monoamniotic pair illustrates the complexity in the interplay of causal factors of these malformations even among monozygotic twins who are assumed to share similar genetic and environmental features. In our case, the occurrence and poor outcome of the malformations was probably potentiated by sub-optimal antenatal care. We noted that with postnatal diagnoses, a better neonatal outcome was difficult to secure even after prompt referral in our setting. Serial obstetric ultrasounds may have helped in early prenatal diagnoses of these malformations as well as prompt counseling of the parents with regards to the high risk of a poor overall outcome and the need for multidisciplinary management.

## Abbreviation

CTEV: congenital talipes equinovarus
